# Retained Catheter in the Aorta

**DOI:** 10.5811/cpcem.2019.7.43425

**Published:** 2019-09-30

**Authors:** Alex Huang, Daniel Quesada, Phillip Aguìñiga-Navarrete, James Rosbrugh, Alexander Wan

**Affiliations:** * Kern Medical Center, Department of Emergency Medicine, Bakersfield, California; † LAC + USC Medical Center, Department of Emergency Medicine, Los Angeles, California

## Abstract

Due to the recent increase in endovascular procedures, retained foreign bodies such as stents and catheters in vasculature have become a common and serious complication. Treatments for these complications vary depending on the acuity and stability of the foreign body in the vessel. We discuss a rare case of an adult found to have an incidental retained umbilical artery catheter in the aorta.

## CASE PRESENTATION

**Image 1:**
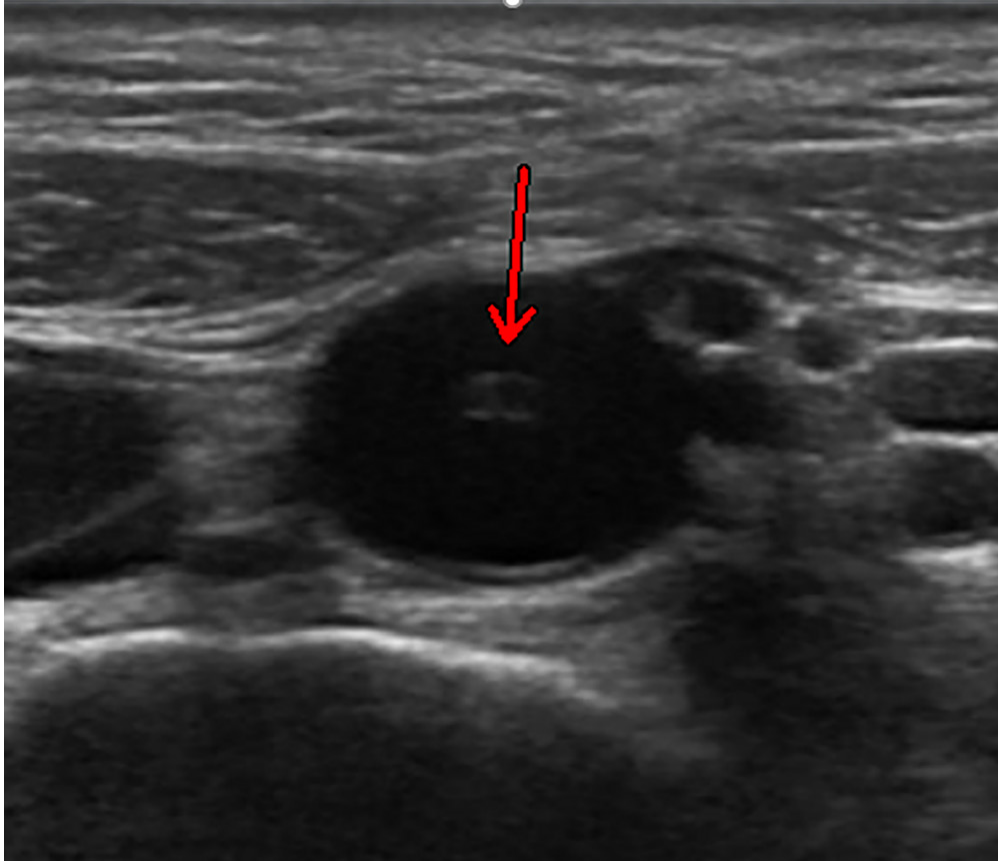
Transverse formal ultrasonographic view of an echogenic tubular structure (arrow) in the aorta.

**Image 2:**
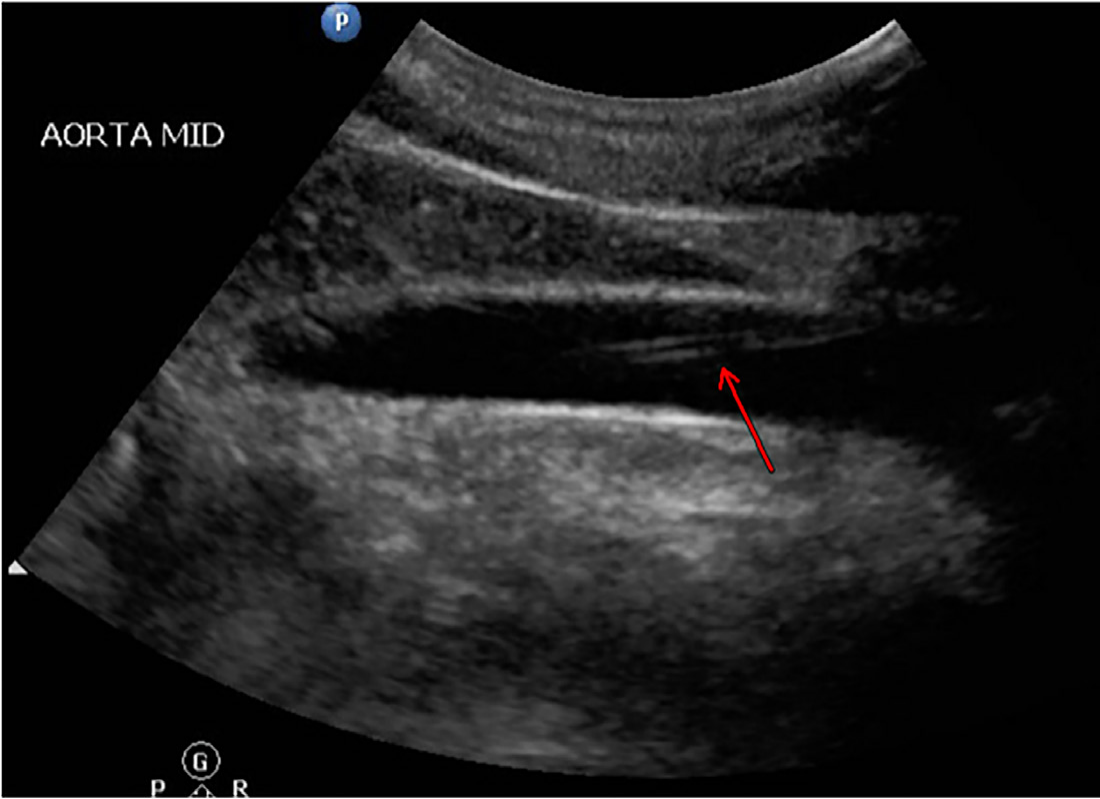
Longitudinal formal utrasonographic view of a 3.9 centimeters (cm), echogenic tubular structure with the distal tip located 1.7 cm from the bifurcation (arrow).

A 25-year-old female with no past medical or surgical history presented to the emergency department with a complaint of intermittent epigastric and abdominal pulsation sensation that she had been experiencing for the prior year. An abdominal aorta ultrasound showed no evidence of aneurysm or dissection, but a 3.9-centimeter, echogenic tubular structure was found in the distal abdominal aorta consistent with a retained catheter fragment (Images 1 and 2). The patient reported that she was born premature at seven months at an outside hospital. Given her history and the ultrasound findings, there was high suspicion for a retained fragment of an umbilical catheter in her aorta. Vascular surgery was consulted. The patient was seen in clinic but was later lost to follow-up before further imaging and treatment.

## DISCUSSION

This case demonstrates an incidental finding of a retained umbilical artery catheter fragment in an adult patient’s aorta. There are several case reports of retained umbilical artery catheter fragments in neonates and infants, which resulted in thrombosis, infection, and embolization.^1^,^2^ This is the first known finding of a retained umbilical artery catheter found in an adult patient. Given the recent increase in endovascular procedures, retained foreign bodies such as stents, coils, guidewires, and catheters in vasculature have become a common and serious complication.^3^ The recommended treatment for acute and nonadherent foreign body is endovascular retrieval, which has a high success rate with minimal mortality. However, in the case of stable intravascular foreign bodies, which are adherent to the vessel wall, the benefits of removal such as decreasing the risk of thrombosis and further migration should be weighed against the risks. In those cases, leaving the foreign body in place is an option.^4^

**CPC-EM Capsule**

What do we already know about this clinical entity?

*Intravascular retained foreign bodies as a result of endovascular procedures can have many complications, including thrombosis and infection*.

What is the major impact of the image(s)?

*This is the first known case of retained umbilical artery catheter fragment found in an adult*.

How might this improve emergency medicine practice?

*Incidental findings of stable intravascular foreign bodies in the emergency department can be managed with observation and referral to vascular surgery*.
